# Integrating Large Language Models into Fluid Antenna Systems: A Survey

**DOI:** 10.3390/s25165177

**Published:** 2025-08-20

**Authors:** Tingsong Deng, Yan Gao, Tong Zhang, Mingjie Shao, Wanli Ni, Hao Xu

**Affiliations:** 1 School of Information Science and Technology, Harbin Institute of Technology, Shenzhen 518055, China; animu2@outlook.com; 2 School of Electronic and Computer Engineering, Peking University, Beijing 100871, China; yangao25@stu.pku.edu.cn; 3 Guangdong Provincial Key Laboratory of Aerospace Communication and Networking Technology, Harbin Institute of Technology, Shenzhen 518055, China; 4 National Mobile Communications Research Laboratory, Southeast University, Nanjing 210096, China; hao.xu@ucl.ac.uk; 5 State Key Laboratory of Mathematical Sciences, AMSS, Chinese Academy of Sciences, Beijing 100190, China; mingjieshao@amss.ac.cn; 6 Department of Electronic Engineering, Tsinghua University, Beijing 100084, China; niwanli@tsinghua.edu.cn

**Keywords:** antenna position optimization, channel estimation, large language model, fluid antenna system

## Abstract

Fluid antenna system (FAS) has emerged as a promising technology for next-generation wireless networks, offering dynamic reconfiguration capabilities to adapt to varying channel conditions. However, FAS faces critical issues from channel estimation to performance optimization. This paper provides a survey of a how large language model (LLM) can be leveraged to address these issues. We review potential approaches and recent advancements in LLM-based FAS channel estimation, LLM-assisted fluid antenna position optimization, and LLM-enabled FAS network simulation. Furthermore, we discuss the role of LLM agents in FAS management. As an experimental study, we evaluated the performance of our designed LLM-enhanced genetic algorithm. The results demonstrated a 75.9% performance improvement over the traditional genetic algorithm on the Rastrigin function.

## 1. Introduction

The fluid antenna systems (FAS) has emerged as a promising reconfigurable antenna technology, attracting significant attention due to its ability to dynamically adjust physical characteristics, such as position, shape, or orientation, to actively adapt to time-varying wireless channel conditions [1,2,3]. Unlike conventional fixed antennas, FAS leverages software-defined control to extend spatial degrees of freedom, significantly enhancing communication performance in complex multipath environments. The growing interest in FAS stems primarily from the stringent demands of future wireless networks for high spectral efficiency, ultra-low latency, and environmental awareness communications. For instance, in dense multi-user environments, FAS can optimize antenna position to mitigate co-channel interference and improve multi-user diversity gain [4,5]. In integrated sensing and communication (ISAC) systems, its flexible spatial reconfiguration capability enables simultaneous optimization of radar detection accuracy and data transmission rates [6]. Consequently, FAS is a key technology for overcoming the limitations of traditional antenna designs, with its development holding substantial significance for next-generation wireless networks.

Despite its advantages, FAS faces several issues that limit its practical applications. The first issue is channel estimation, which demands extensive resource-intensive measurements. The second issue is antenna position optimization, where the solution must achieve high performance while remaining computationally efficient. The third issue lies in FAS network simulation, which must accurately replicate real-world conditions while enabling rapid evaluation and dynamic scenario reconfiguration. These issues have attracted significant attention, yet they remain insufficiently addressed.

Large language models represent a class of deep learning-based artificial intelligence systems characterized by their massive parameter scale and multimodal comprehension capabilities, enabling them to perform diverse tasks such as natural language processing, computer vision, behavioral understanding, and content generation. The advent of LLMs has precipitated transformative advances across multiple disciplines, including wireless communications [7,8,9,10], autonomous driving [11,12,13], and more. In wireless communications, LLM has envisioned being applied into large-scale multi-input multi-output (XL-MIMO) [7], reconfigurable intelligent surface (RIS) [8], ISAC [9], and radio map generation [10], demonstrating significant performance improvements over traditional approaches. For instance, Dai et al. [7] applied LLM to optimize near-field XL-MIMO communications for the low-altitude economy, addressing key challenges in beam focusing and spectrum efficiency maximization through a novel LLM-based scheme that outperforms existing benchmarks. Xu et al. [8] proposed an LLM-enhanced RIS framework for 6G internet of vehicles (IoV), leveraging LLM’s analytical capabilities to dynamically optimize RIS configurations based on real-time vehicular data, thereby achieving energy-efficient and reliable communications while overcoming the challenges of vehicular environment dynamics, with simulations validating its superior performance. Li et al. [9] investigated a network of unmanned aerial vehicles (UAVs) with ISAC capabilities, formulating a multi-objective optimization problem to balance communication and sensing performance and proposed an LLM-enhanced evolutionary algorithm that outperforms baseline methods in achieving optimal trade-offs. Quan et al. [10] developed an automated LLM agent framework for radio map generation and wireless network planning, which significantly minimizes manual intervention.

Addressing these fundamental FAS challenges and unlocking the system’s potential, this paper systematically investigates the potential to revolutionize FAS through the integration of LLM. We examine the following: LLM-enhanced channel estimation for improved signal characterization, LLM-driven antenna position optimization for dynamic reconfiguration, LLM-based network traffic simulation for realistic performance evaluation, and specialized LLM agents for autonomous FAS management. The challenges and future research opportunities in applying LLM to FAS are finally highlighted. Furthermore, motivated by the idea from MindEvolution [14], we designed an LLM-enhanced genetic algorithm and evaluated its performance on the Rastrigin function and a three-path FAS channel function. Our experimental results include a 75.9% performance improvement over the traditional genetic algorithm on the Rastrigin function. For the three-path FAS channel, both the LLM-enhanced and traditional genetic algorithms demonstrated comparable performance, each approaching theoretical optimality. We attribute this improvement to the LLM’s ability to perform more effective crossover and mutation by leveraging its knowledge of the Rastrigin function. As for the three-path FAS channel function, the results may reflect the presence of numerous local optima with comparable performance. Our code is available at https://github.com/TingsongDeng/LLM-GAs.git (accessed on 8 August 2025).

**Figure 1:**
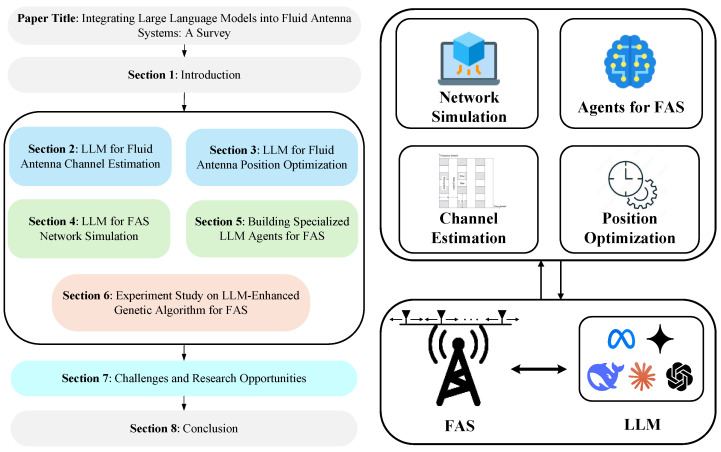
The organization of this paper.

The remainder of this paper is organized as follows: Section 1 introduces the background of FAS and the challenges, along with potential applications, of LLM in FAS deployment. Section 2 provides a detailed analysis of LLM-based fluid antenna channel estimation. Section 3 focuses on LLM-driven antenna position optimization strategies, with four possible solutions being proposed and a comprehensive comparison of their performance and requirements being presented. Section 4 highlights LLM-assisted FAS network simulation techniques. Section 5 introduces LLM agents and discusses several potential application schemes for addressing specific problems that may arise during FAS deployment. Additionally, Section 6 incorporates LLM into the crossover and mutation operations of genetic algorithms, experimental tests were conducted on both the Rastrigin function and the FAS channel function. Challenges and research opportunities are discussed in Section 7, while conclusions are provided in Section 8. An illustration of this organization is given in Figure 1.

## 2. LLM for Fluid Antenna Channel Estimation

In FAS, channel estimation is crucial as it identifies the high-performance antenna positions (“good” positions) while avoiding mediocre ones (“bad” positions). Accurate channel state information enables FAS to dynamically switch to optimal ports, significantly outperforming traditional fixed antenna arrays. However, since FAS typically employs a large number of ports (e.g., 256 or continuous ports) but can only measure a few at a time (e.g., four antennas due to hardware constraints), channel estimation demands substantial resources. In addition, unlike conventional MIMO systems, FAS features extremely small port spacing, resulting in high-dimensional channels with strong spatial correlation. To address this challenge, state-of-the-art FAS channel estimation research can be divided into two categories. The first one employs numerical methods, including linear minimum mean square error (LMMSE) [15], compressed sensing [16], successive Bayesian learning [17], sparse Bayesian learning [18], and orthogonal matching pursuit (OMP) [19]. The second one is leveraging neural networks, including dedicated sub-networks [20], asymmetric graph masked autoencoder [21], conditional generative adversarial network [22], and diffusion model [23]. However, existing methods still struggle to achieve accurate FAS channel estimation with limited samples.

The LLM introduces novel possibilities for FAS channel estimation by effectively modeling complex real-world signal propagation, enabling few-shot sampling and accurate prediction. Unlike conventional techniques, LLM adapts dynamically to varying channel conditions and leverages contextual data (e.g., geographic and environmental factors) to improve accuracy. Additionally, LLM can optimize signal training patterns through data-driven insights. A key enabler is tokenizing channel characteristics, which allows LLM to process FAS estimation efficiently. As demonstrated in [24], this approach significantly reduces estimation overhead via predictive modeling.

At present, research on leveraging LLM to improve fluid antenna channel estimation remains limited, with notable contributions from [25,26]. In [25], the authors explore LLM-enhanced channel estimation for low-earth orbit (LEO) satellite Internet of Things (IoT) networks. By employing a low-rank-adaptation (LoRA)-optimized LLaMA-3 model to process compressed channel representations, their approach achieves a 10 dB normalized mean squared error (NMSE) improvement over traditional predictors such as LSTM and GRU. Meanwhile, in ref. [26], the authors introduce a novel framework addressing two key challenges in massive MIMO channel prediction. First, it resolves the modality mismatch between linguistic knowledge in pre-trained LLMs and channel state information (CSI) via a cross-modal alignment module. This module projects CSI features into the LLM’s semantic space using principal-component-analysis (PCA)-reduced word embeddings and similarity-based semantic prompts. Second, to mitigate computational inefficiency, the authors propose CSI-ALM-Light, a lightweight variant distilled from CSI-ALM through attention matrix optimization. With only 0.34 million parameters, CSI-ALM-Light matches the performance of its larger counterpart while offering practical deployability.

## 3. LLM for Fluid Antenna Position Optimization

Fluid antenna position optimization is a critical issue for FAS. In particular, a suitable antenna position will greatly enhance FAS performance, as different antenna positions may have significant distinctions. Currently, research on fluid antenna position optimization can be categorized into three main approaches. The first approach employs convex and nonconvex optimization methods, such as gradient descent [27], alternating optimization (AO) [28], successive convex approximation (SCA) [29], and majorization–minimization (MM) [30]. These methods seek tractable approximations of the original problem formulation, typically yielding convergent solutions with moderate computational overhead. The second approach utilizes evolutionary algorithms, including standard particle swarm optimization (PSO) [31], multi-velocity particle swarm optimization (MVPSO) [32], and genetic algorithms [33]. The third approach leverages deep reinforcement learning (DRL) techniques, such as advantage actor-critic (A2C) [34], team-inspired DRL [35], and multi-agent deep deterministic policy gradient (MADDPG) [36].

Nevertheless, each of these methods exhibits distinct advantages and limitations. Convex and nonconvex optimization techniques achieve the fastest convergence rates but are prone to becoming trapped in stationary points. In contrast, evolutionary algorithms demonstrate superior performance by thoroughly exploring the objective function’s landscape, albeit at the cost of significantly slower convergence. DRL typically delivers the best overall performance; however, it demands substantial computational resources, extensive training time, and large datasets.

LLM-driven optimization holds substantial promise for overcoming the limitations of traditional optimization methods. By leveraging the advanced reasoning capabilities and computational power of LLMs, recent work (e.g., [37]) has demonstrated their effectiveness in solving optimization problem. In the following section, we review the state-of-the-art LLM-driven optimization approaches relevant to FAS.

### 3.1. LLM as a Black-Box Optimization Search Model

LLM can explore solution spaces through generative search without requiring explicit problem structure modeling. In particular, LLM can generate candidate solutions without mathematical formulation. Furthermore, LLM can inherently avoid generating infeasible solutions that violate physical laws or common sense constraints.

An example is DeepMind’s Optimization by PROmpting (OPRO) framework [38], which introduces a specialized architecture for mathematical optimization through natural language processing. The framework uniquely transforms optimization objectives into natural language prompts while systematically incorporating historical solving attempts into its reasoning process. By generating new candidate solutions based on previous optimization trajectories and continuously updating its meta-prompt vocabulary with these results, the system creates a self-improving loop that progressively refines the LLM’s problem-solving strategy. This iterative approach enables the model to learn directly from its optimization history while maintaining the flexibility to adapt its search direction based on accumulated experience. However, this approach still exhibits significant limitations:

- **No convergence guarantee**. Prompt engineering and parameter tuning critically affect the solution quality of LLM-based black-box optimization.
- **Susceptibility to local optima**. The iterative generation process often converges to suboptimal solutions and exhibits instability in continuous variable optimization.
- **High computational cost**. Each optimization iteration requires a complete forward pass of the LLM, making the process substantially less efficient than conventional optimization algorithms.

### 3.2. LLM-Guided Deep Reinforcement Learning

Deep reinforcement learning (DRL) employs neural networks to approximate value functions or policy functions or both functions, thereby addressing the curse of dimensionality in high-dimensional state or action spaces. The end-to-end learning characteristic of DRL enables direct mapping from raw inputs to action outputs, eliminating the need for complex feature engineering and modular design in traditional RL and thus streamlining the process. Additionally, DRL’s nonlinear approximation capability allows it to represent complex strategies, far surpassing the linear functions or tabular methods used in traditional RL. Despite its advantages, DRL inevitably has four weaknesses:

- **Low sample efficiency**. DRL demands extensive environmental interaction samples, resulting in high training costs and challenges for real-world physical systems.
- **Difficulty in learning effectively under sparse rewards**. DRL struggles to learn efficiently in sparse reward scenarios.
- **High sensitivity to hyperparameters**. Extensive experimentation is needed for tuning, and training can be unstable due to unsuitable hyper-parameters.
- **Limited generalization in highly dynamic environments**. DRL generalization performance may drop sharply when environmental conditions change significantly.

To combat the above weakness of DRL, LLM-guided DRL aims at leveraging LLM to enhance the training efficiency, generalization capability, and interpretability of DRL. LLM-guided DRL brings the semantic understanding, task decomposition, and reasoning abilities of LLM into DRL, which excels at the following.

- **LLM-generated data for enhancing sample efficiency**. LLMs can provide prior knowledge by generating reasonable initial policies or sub-goals based on existing knowledge, thereby reducing random exploration and accelerating DRL convergence. Additionally, LLMs can generate data to simulate expert trajectories. For example, Wang et al. [39] use LLM to generate construction plans while DRL manages execution, drastically cutting training time. For another example, Zhu et al. [40] proposed a novel approach called LAMARL (LLM-aided multi-agent reinforcement learning), which utilizes LLMs to generate prior policies and achieves an average 185.9% improvement in sample efficiency. Most recently, Du et al. [41] introduced RLLI, integrating RL with LLM interaction. It employs LLM-generated feedback as RL rewards to enhance convergence and uses LLM-assisted optimization to improve sample efficiency by reducing redundant computations.
- **LLM for handling sparse rewards**. LLM can automatically generate dense reward signals by designing intermediate rewards based on task descriptions to guide the learning process. For example, Ma et al. [42] proposed Eureka, which leverages LLMs to automate and optimize reward functions for DRL tasks.
- **LLM-based hyperparameter optimization**. LLM-based hyperparameter optimization automates the tuning process for DRL, efficiently discovering optimal configurations. For example, Liu et al. [43] proposed a framework named AgentHPO, which utilizes LLM agents to automate the hyperparameter optimization process.
- **LLM-enhanced generalization**. LLM-guided DRL improves generalization by decomposing tasks and facilitating transfer learning. It breaks complex tasks into familiar subtasks, enabling policy reuse and zero-shot or few-shot adaptation through natural language understanding. For example, Ahn et al. [44] proposed SayCan, an innovative framework that leverages LLMs to interpret high-level instructions and decompose them into executable subtasks, which are then sequentially executed by DRL agents.

### 3.3. LLM-Guided Evolutionary Algorithms

Evolutionary algorithms are a class of biologically inspired, population-based optimization methods. As a key branch of computational intelligence, they excel at solving complex optimization problems by mimicking natural evolutionary processes, such as selection, mutation, and recombination. However, despite their advantages in addressing certain optimization challenges, evolutionary algorithms still exhibit notable weaknesses:

- **Trapped into local optima**. Traditional evolutionary algorithms, such as genetic algorithms, often become trapped in suboptimal solutions due to limited population diversity or deceptive fitness landscapes.
- **Curse of dimensionality**. Search efficiency declines exponentially as problem dimensionality increases, requiring specialized handling for problems exceeding thousands of dimensions. Certain optimization processes may require tens of thousands of fitness evaluations, leading to high computational expenses.
- **Constraint handling difficulties**. Formalizing expert knowledge into fitness function constraints proves challenging.

To combat the above weaknesses, LLM-guided evolutionary algorithms fundamentally reposition LLMs from potential direct solvers to sophisticated design assistants that synergistically combine domain-specific knowledge with advanced analytical capabilities. This approach demonstrates the following distinct advantages over conventional evolutionary algorithms.

- **Helps to escape from local optima.** By leveraging LLMs’ capabilities in crossover, mutation, and other exploration operations, LLM-enhanced evolutionary algorithms demonstrate improved ability to escape local optima. For example, as reported in [14], Deepmind developed MindEvolution, which adapts a genetic algorithm to solve natural language problems using LLM to perform crossover and mutation in text-based solutions.
- **LLM-powered dimensionality reduction**. LLMs leverage natural language to describe complex individuals, mapping high-dimensional spaces into low-dimensional semantic representations and thus overcoming the limitations of traditional numerical encoding. The introduction of LLM can break through the efficiency bottleneck of hyperparameter optimization in evolutionary algorithms. For example, Romera et al. [45] proposed the FunSearch method, which combines LLM and evolutionary algorithms to compress the search space, achieving significant breakthroughs in solving problems such as CapSet. For another example, Hameed et al. [46] proposed an innovative approach leveraging ChatGPT-3.5 and Llama3 to generate optimized particle positions and velocity suggestions, which replace underperforming particles in PSO, thereby reducing model evaluation calls by 20–60% and significantly accelerating convergence.
- **LLM-based semantic constraint processing**. LLM can automatically transform expert-described fuzzy constraints into computable mathematical expressions while dynamically adjusting constraint weights and integrating multimodal constraints, thereby eliminating the need for manual constraint design in traditional evolutionary algorithms. For example, Shinohara et al. [47] proposed a method called LMPSO. For constraint handling, this method enables users to directly specify constraints in natural language, with the LLM automatically adhering to these constraint requirements when generating solutions. Additionally, through a meta-prompt mechanism, it supports dynamic adjustment of constraints or heuristic rules during the optimization process.

### 3.4. AlphaEvolve-like Approach: LLM as Solution Program Generator and Critic

Google DeepMind proposed AlphaEvolve in May 2025, a programming agent that combines LLMs with evolutionary algorithms [48]. This agent integrates generative models (e.g., policy networks) and critics (e.g., value networks) to iteratively optimize solutions. This architecture can be adapted to LLM application scenarios, enabling them to serve dual roles as both solution generators and quality evaluators, thereby constructing a self-optimizing closed-loop system.

**Figure 2:**
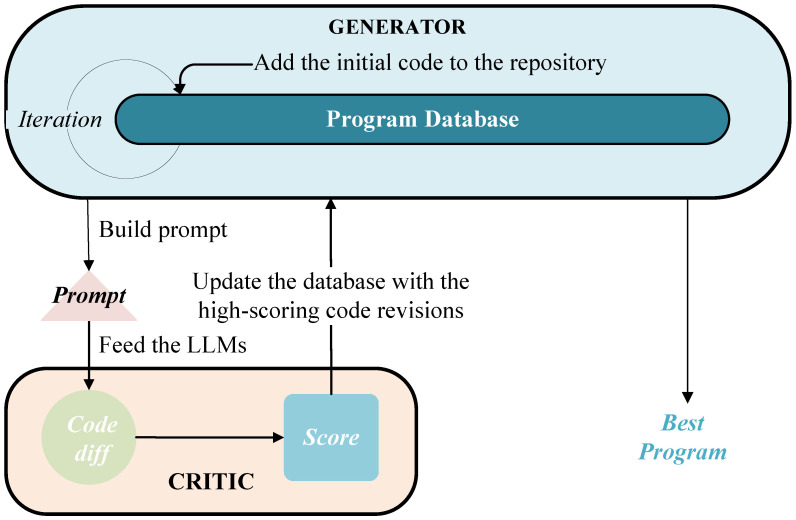
LLM as solution program generator and critic.

The core of this AlphaEvolve-like methodology lies in leveraging LLMs as a collaborative system of solution generators and critics, as illustrated in Figure 2. The LLM-based optimization follows an iterative generate–evaluate–refine cycle. First, as a policy network, it generates diverse candidate solutions through multi-path reasoning, with domain-specific fine-tuning improving solution relevance. Then, as a critic, it evaluates solutions across key metrics such as correctness, efficiency, and robustness. The system retains top solutions, refines or discards others, and produces improved variants, repeating until meeting quality standards. Compared to conventional LLM inference, this evolutionary approach offers several distinct advantages:

- **Iterative solution refinement**. The optimization solution evolves through iterations, mitigating early-stage error accumulation and outperforming single-pass self-optimization in closed-loop systems.
- **Feedback learning mechanism**. This AlphaEvolve-like approach can demonstrate effective learning from performance feedback.
- **Creativity–rigor Synergy**. This approach successfully integrates the creative generation of optimization solutions with rigorous evaluation protocols.

### 3.5. Comparison and Discussion

When FAS is deployed in practice, real-time and solution quality should be the top priorities. In additional, lower memory and floating-point operations (FLOPs) requirements, along with a simple design, are desirable. Real-time performance is crucial, as capturing channel dynamics requires rapid processing. In this regard, a neural network trained via LLM-guided DRL (LLM-DRL) undoubtedly offers fast inference speeds, meeting real-time demands. Furthermore, if evolving from a simple yet fast baseline solution, such as gradient descent, LLM acting as both a solution generator and critic (LLM-Alpha) has the potential to produce a significantly better program. Solution quality directly governs FAS effectiveness. Here, LLM-DRL- and LLM-guided evolutionary search (LLM-EA) likely perform similarly, as both strategies pursue near-complete search space coverage. The LLM-Alpha, which searches for optimal programs through extensive computation, demands significantly higher FLOPs and memory resources than do other methods due to its exhaustive search requirements. The detailed comparative results are presented in Table 1, where we estimate the number of stars by our knowledge. For FAS deployment, we recommend the LLM-DRL, as its trained model can deliver satisfactory performance with efficient real-time execution.

## 4. LLM for FAS Network Simulation

With the rapid advancement of FAS, the demand for accurate and efficient traffic simulation tailored to these new network architectures is steadily increasing [49]. Traditional traffic simulation methods are often limited in their ability to model the fine-grained, dynamic nature of FAS networks. These methods struggle to capture complex spatial-temporal effects and the diverse behaviors of users in realistic environments, making them less effective in FAS scenarios [50]. Statistical models such as Poisson and Markov processes also fail to reflect the high variability of traffic patterns in real FAS networks. LLM has demonstrated remarkable capabilities in data modeling, contextual understanding, task planning, and cross-modal reasoning for network simulation [51]. Integrating LLM into FAS network traffic simulation introduces a novel paradigm with significant advantages in the following aspects:

- **Human-like behavior modeling**. LLMs are capable of simulating human behaviors in a highly realistic manner, thereby generating network traffic patterns that closely resemble real-world conditions. Human interactions with networks are typically personalized and diverse, involving variations in access time, usage habits, application types, and traffic fluctuations. In complex environments, such behaviors are multi-modal and context-dependent rather than uniform. LLMs can capture such dynamics, learn behavioral diversity and patterns from real-world data, and generate adaptive, anthropomorphic traffic that evolves over time and context.
- **Strong adaptivity to new scenarios**. With the integration of LLMs, FAS systems can adapt in real-time to changing network conditions, such as congestion or signal degradation, by modifying user behaviors such as adjusting video quality or pausing downloads. While LLMs generally exhibit some inference latency, they can still simulate dynamic network environments by leveraging pre-trained models and responding quickly to shifts in network conditions. Achieving true real-time adaptability would require specialized hardware or further optimization, such as model compression, to enhance inference speed and efficiency.
- **Controllable and interpretable traffic generation**. Unlike traditional black-box models, LLMs offer explicit control over simulation parameters through prompt engineering, such as quality of service (QoS) requirements and hardware limitations, improving the transparency and repeatability of traffic generation.
- **Reduced complexity and improved simulation efficiency**. By converting complex simulation tasks into high-level natural language descriptions, LLMs streamline the simulation process, reducing computational burden and improving the speed of generating large-scale simulations.

For example, the recently proposed TrafficLLM framework in [52] employs a fine-tuning mechanism that enables LLMs to generate network traffic based on natural language instructions. This framework outperforms traditional models by improving detection task F1 scores (a measure of a model’s accuracy in classification tasks, considering both precision and recall) by over 10% and reducing the Jensen–Shannon divergence between generated and real traffic by approximately 39.3%. Similarly, the ChatSim framework in [53] utilizes multiple LLM agents working in collaboration to transform natural language descriptions into executable traffic scripts. This system showcases how LLMs can handle complex, multimodal scenarios and generate high-fidelity traffic that aligns with dynamic FAS demands. The flexibility and realism provided by ChatSim highlight the potential of LLMs to revolutionize traffic simulation in FAS environments.

Incorporating LLMs into FAS network traffic simulation not only enhances realism and efficiency but also enables adaptive modeling and real-time optimization. As LLM technology advances, it is expected to drive the development of generative network simulators, creating self-evolving systems that optimize network configurations and operations. This will play a pivotal role in the future of intelligent communication systems, especially in the context of ISAC and 6G networks.

## 5. Building Specialized LLM Agents for FAS

LLM agents now serve as the core in autonomous systems. These agents combine three critical functions—task planning through goal decomposition and strategy optimization, adaptive memory for both short-term context and long-term knowledge, and tool integration—to extend capabilities beyond pure language processing, enabling everything from software coding to hardware control. In the field of LLM Agents, there exist three fundamental patterns:

- **Tool use and planning pattern**. The model employs a multi-task coordination and strategic decomposition mechanism to break down objectives into sequentially executed subtasks, dynamically optimizing task priorities through real-time feedback. By integrating external tools, it effectively mitigates hallucination issues and knowledge obsolescence, enabling efficient handling of complex problems. For example, The LLM-Planner proposed by Song et al. [54] employs a hierarchical planning and dynamic re-planning coordination framework, significantly enhancing agent decision-making capabilities in complex scenarios. Experimental results on the ALFRED benchmark demonstrate the system’s strong generalization ability and dynamic environment adaptation under few-shot conditions.
- **ReAct pattern**. The ReAct pattern enables cognitive–behavioral unity through its “reason–plan–act–optimize” loop, creating self-correcting intelligence via dynamic task processing. This paradigm moves beyond one-way generation, achieving “knowledge–action unity” through environmental interaction and continuous optimization. For example, Wang et al. [55] proposed an LLM-based agent framework that generates hyperparameter optimization strategies through a multi-path reasoning mechanism while dynamically integrating environmental feedback for strategy adaptation. The framework employs an enhanced WS-PSO-CM algorithm for hyperparameter evaluation, establishing an advanced ReAct pattern. Experimental results demonstrate that compared to conventional manual heuristic methods, this framework achieves a remarkable 54.34% performance improvement in hyperparameter optimization tasks, exhibiting significant optimization effectiveness.
- **Multi-agent pattern**. The multi-agent establishes a complex task-processing system through multi-agent collaboration. Task execution in multi-agent systems can be parallelized, unlike in single-agent systems where it is sequential, thereby improving efficiency and reducing latency. For example, Lowe et al. [56] proposed the MADDPG algorithm, which introduces a novel centralized training with decentralized execution (CTDE) paradigm. During training, each agent’s critic network receives the concatenated observations and actions of all agents as input, enabling the learning of global coordination patterns while maintaining policy independence. For execution, agents rely solely on local observations to make autonomous decisions, achieving full decentralization. Experimental results demonstrate this framework’s superior capability in addressing the non-stationarity challenges inherent in multi-agent reinforcement learning (MARL) environments.

The application of LLM agents in FAS enables automated adjustment, significantly outperforming traditional human-experience-dependent design approaches while adapting to rapidly changing communication environments. By real-time monitoring of CSI and interference conditions, the agent can autonomously optimize antenna configurations to dynamically enhance signal quality without relying on fixed rules or manual intervention. Its data-driven decision-making capability overcomes the inherent limitations of human expertise and response latency in conventional design while also improving energy efficiency, coverage, and interference mitigation.

## 6. Experiment Study on LLM-Enhanced Genetic Algorithm for FAS

### 6.1. System Model and Problem Formulation

The considered system has one transmitter and one receiver, where both of them are equipped with a single fluid antenna. According to the field response model [57], the channel between the transmitter and the receiver can be given as follows:

(1) $$h\left(v,u\right)=f^{H}\left(v\right)\Sigma g\left(u\right),$$

where $f\left(v\right)={\left[e^{j\frac{2\pi }{\lambda }\rho_{1}^{r}\left(v\right)},\ldots ,e^{j\frac{2\pi }{\lambda }\rho_{L}^{r}\left(v\right)}\right]}^{T}\in C^{L\times 1}$ denotes the field response vector at the receiver, and $v=(x^{r},y^{r},z^{r})$ denotes the position of receive antenna; $\Sigma =\mathrm{diag}\left\{\sigma_{1},\ldots ,\sigma_{L}\right\}\in C^{L\times L}$ denotes the path response matrix with *L* paths and $\sigma_{j}∼\mathrm{CN}\left(0,g_{0}d^{-\alpha }/L\right)$, j∈[L]; and $g\left(u\right)={\left[e^{j\frac{2\pi }{\lambda }\rho_{1}^{t}\left(u\right)},\ldots ,e^{j\frac{2\pi }{\lambda }\rho_{L}^{t}\left(u\right)}\right]}^{T}\in C^{L\times 1}$ denotes the field response vector at the transmitter, and $u=(x^{t},y^{t},z^{t})$ denotes the position of transmit antenna. In particular, $\rho_{j}^{r}\left(v\right)=x^{r}\eta_{j}^{r}+y^{r}\beta_{j}^{r}+z^{r}\omega_{j}^{r}$ denotes the phase for path j∈[L] from the receiver side, where with pitch angle $\theta_{j}^{r}\in \left[-\pi /2,\pi /2\right]$ and azimuth angle $\phi_{j}^{r}\in \left[-\pi /2,\pi /2\right]$ for path *j* from the receiver side, we have

(2a) $$\begin{array}{ccc} & & \eta_{j}^{r}=\cos\theta_{j}^{r}\cos\phi_{j}^{r}, \end{array}$$

(2b) $$\begin{array}{ccc} & & \beta_{j}^{r}=\cos\theta_{j}^{r}\sin\phi_{j}^{r}, \end{array}$$

(2c) $$\begin{array}{ccc} & & \omega_{j}^{r}=\sin\theta_{j}^{r}. \end{array}$$

Moreover, $\rho_{i}^{t}\left(v\right)=x^{t}\eta_{i}^{t}+y^{t}\beta_{i}^{t}+z^{t}\omega_{i}^{t}$ denotes the phase for path i∈[L] from the transmitter side, where with pitch angle $\theta_{i}^{t}\in \left[-\pi /2,\pi /2\right]$ and azimuth angle $\phi_{i}^{t}\in \left[-\pi /2,\pi /2\right]$ for path *i* from the transmitter side, we have

(3a) $$\begin{array}{ccc} & & \eta_{i}^{t}=\cos\theta_{i}^{t}\cos\phi_{i}^{t}, \end{array}$$

(3b) $$\begin{array}{ccc} & & \beta_{i}^{t}=\cos\theta_{i}^{t}\sin\phi_{i}^{t}, \end{array}$$

(3c) $$\begin{array}{ccc} & & \omega_{i}^{t}=\sin\theta_{i}^{t}. \end{array}$$

We aim to maximize the rate of this point-to-point FAS by optimizing the position of fluid antennas at the transmitter and receiver. This problem is mathematically formulated as

(4a) $$\begin{array}{cc} P0:\;\;\;\;\max_{v,u} & \log_{2}\left(1+\frac{{P|h\left(v,u\right)|}^{2}}{\sigma^{2}}\right) \end{array}$$

(4b) $$\begin{array}{cc} s.t. & v\in C^{r},\;\;u\in C^{t}, \end{array}$$

where *P* denotes transmit power, $\sigma^{2}$ denotes the variance of additive white Gaussian noise (AWGN), $C^{r}$ denotes the receive antenna movable region, and $C^{t}$ denotes the transmit antenna movable region. Note that Problem P0 is a non-convex problem.

### 6.2. Traditional Genetic Algorithm

Genetic algorithm belongs to evolutionary optimization techniques inspired by natural selection and genetics [58]. Genetic algorithm works by maintaining a population of candidate solutions, which evolve over generations through selection, crossover, and mutation. This algorithm is particularly useful for solving complex, non-linear, and non-convex problems. Specifically, the process begins by initializing a population with random or heuristic-based individuals, each encoded as chromosomes (binary, real-valued, etc.). A fitness function evaluates each solution’s quality, guiding selection, where high-fitness individuals are more likely to reproduce. Selected parents undergo crossover, exchanging genetic material to produce offspring, while mutation introduces small random changes to maintain diversity. This cycle repeats until a termination condition (e.g., max generations or convergence) is satisfied.

### 6.3. LLM-Enhanced Genetic Algorithm

**Figure 3:**
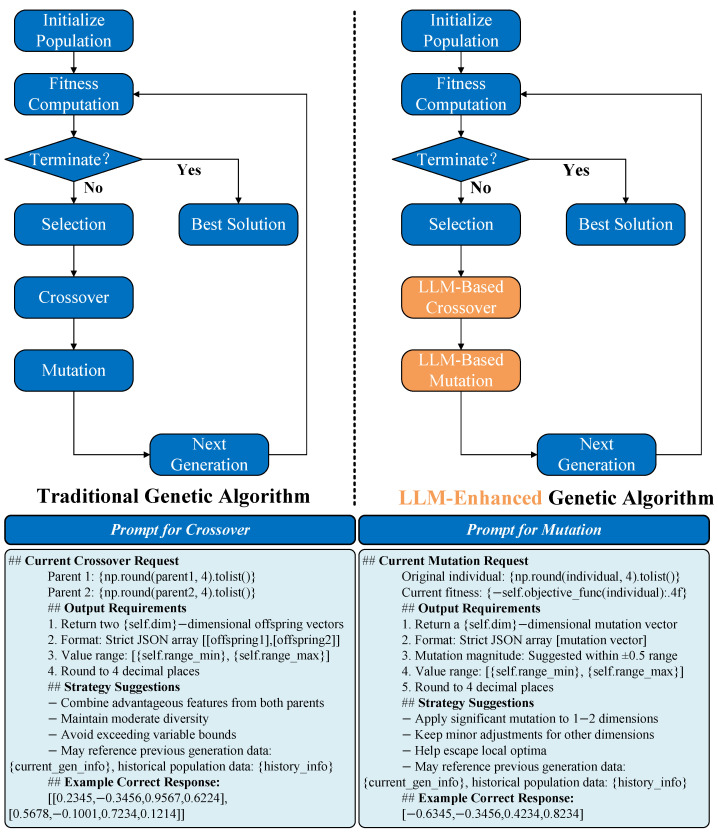
Comparison of traditional genetic algorithm and LLM-enhanced genetic algorithm.

Motivated by the idea introduced in [14], we designed an LLM-enhanced genetic algorithm that leverages large language models to redefine evolutionary operations. Instead of traditional crossover and mutation, LLMs generate and refine candidate solutions by interpreting contextual prompts, enabling more intelligent exploration of the search space. We compare the traditional genetic algorithm with the proposed LLM-enhanced genetic algorithm in Figure 3. It can be seen that crossover and mutation operations in the traditional genetic algorithm are replaced by LLM in the LLM-enhanced genetic algorithm. The LLM is aware of the objective function and constraints. Along with historical optimization knowledge, LLM can perform crossover and mutation superior to traditional randomized crossover and mutation. This point was validated by the experiments described below.

### 6.4. Experiment Study

To examine the performance of the LLM-enhanced genetic algorithm, we performed experiments on two objective functions. We implemented our LLM-enhanced genetic algorithm on the DeepSeek R1. First of all, we considered the celebrated Rastrigin function, which is a classic multimodal optimization test function, commonly used to evaluate the performance of optimization algorithms (such as genetic algorithms and particle swarm optimization). It features a large number of local minima within the search space, with the global minimum located at the origin (or a specified offset point), making it well-suited for testing an algorithm’s global search capability. The Rastrigin function is given by the following:

(5) $$f\left(x\right)=10n+\sum_{i=1}^{n}\left(x_{i}^{2}-10\cos\left(2\pi x_{i}\right)\right).$$

**Figure 4:**
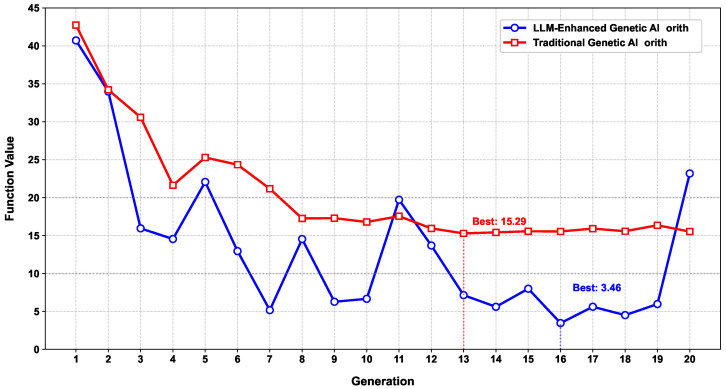
Genetic algorithm performance comparison on the Rastrigin function (5).

Next, we tried to minimize the Rastrigin function with $x=(x_{1},\ldots ,x_{5})$ using a population size of 30. As shown in Figure 4, the LLM-enhanced genetic algorithm exhibited a significant advantage over the traditional genetic algorithm. More specifically, LLM-enhanced genetic algorithm and traditional genetic algorithm achieved a minimum of 3.4642 and 14.3703, respectively, achieving a 75.9% improvement. The performance gain may come from the following: (1) The traditional genetic algorithm relies on fixed-probability mutation and crossover rules, which often lead to local optima. In contrast, LLM-enhanced algorithms dynamically analyze population diversity and fitness distribution to intelligently adjust mutation rates or design superior crossover schemes. (2) The traditional genetic algorithm relies on numerical perturbations (e.g., Gaussian mutation) that lack directional guidance. The LLM-enhanced method leverages natural language understanding to interpret problem constraints and objectives, generating semantically valid candidate solutions.

(6) $$\begin{array}{cc} \left|h(y^{t},z^{t},y^{r},z^{r})\right| & =\left|\sum_{i=1}^{3}A_{i}\exp\left(j\omega \gamma_{i}\left(y^{t},z^{t},y^{r},z^{r}\right)\right)\right|,\\ \mathrm{where}\;\omega & =1256.6,\\ A & =[0.634,\;0.1768,\;0.1768],\\ \gamma_{1} & =0.1545+0.4755y^{t}+0.8660z^{t}+0.3536y^{r}+0.7071z^{r},\\ \gamma_{2} & =0.6371+0.3068y^{t}+0.7071z^{t}+0.3510y^{r}+0.5878z^{r},\\ \gamma_{3} & =0.6123+0.6123y^{t}+0.5z^{t}+0.2939y^{r}+0.8660z^{r}. \end{array}$$

**Figure 5:**
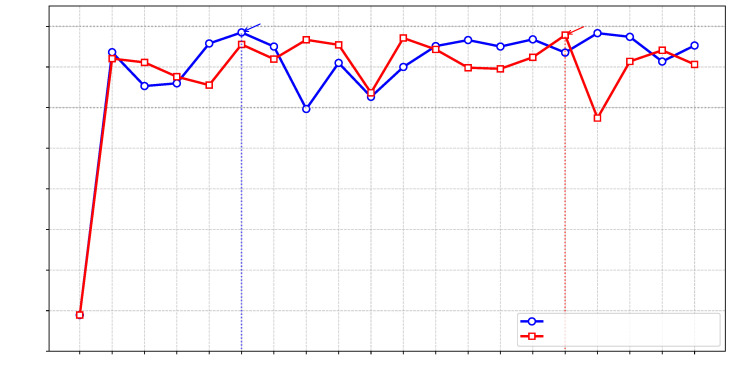
Genetic algorithm performance comparison on the three-path FAS channel function (6).

Finally, we shifted our focus to FAS and using a population size of 10. As a representative, according to the field response model, the three-path channel was considered, as shown in (6). The function has a theoretical upper bound, given as $\left|h(y^{t},z^{t},y^{r},z^{r})\right|=\left|\sum_{i=1}^{3}A_{i}\exp\left(j\omega \gamma_{i}\right)\right|\leq \sum_{i=1}^{3}\left|A_{i}\right|=0.9856$, where the triangle inequality is applied. The details parameters of this three-path channel are given in Table 2. Figure 5 shows that the LLM-enhanced genetic algorithm achieved performance levels similar to those of the traditional genetic algorithm, with both nearing theoretical optimality. We may attribute this to the structure of the objective function (6), which can be transformed into a summation of multiple cosine functions, leading to numerous local optimal points with comparable performance.

## 7. Challenges and Research Opportunities

Despite the promising potential of LLMs in enabling FAS, several significant challenges remain that need to be addressed before their full integration into real-world systems. These challenges span technical, practical, and conceptual domains, and overcoming them will be essential for realizing the full potential of LLM-powered FAS:

- **Fusion and alignment of multi-modal data**. FAS environments involve a variety of heterogeneous information sources, such as antenna configurations, CSI, environmental semantics, and user behavior patterns. However, most LLMs are primarily trained on text data, making it difficult to align and integrate multimodal inputs (e.g., numerical, visual, and semantic data). Developing unified frameworks capable of processing and aligning these diverse data sources remains a significant technical challenge [59]. Bridging this gap will require the development of sophisticated multimodal learning systems that can handle both structured data (e.g., CSI) and unstructured data (e.g., user behavior) seamlessly.
- **Low-latency inference and computational efficiency**. FASs often operate in edge environments with limited computational resources and stringent latency requirements. Current LLMs are computationally expensive and exhibit high inference latency, limiting their real-time adaptability in dynamic FAS scenarios. Lightweight approaches such as LoRA and mixture-of-experts (MoE) models have shown promise in improving efficiency, but further research is required to optimize these models for low-latency inference in resource-constrained environments [60]. The development of efficient model architectures that can provide high-performance inference with minimal computational overhead will be crucial for enabling real-time FAS applications.
- **Security and interpretability**. While LLM-driven optimization in FAS offers greater flexibility and autonomy, the black-box nature of these models presents significant risks, especially in mission-critical communication scenarios [61]. Unpredictable behaviors could arise if LLMs are left uncontrolled, leading to potential instability in system performance. Therefore, building interpretable, constraint-aware LLM controllers that allow users to understand and predict model decisions is essential for ensuring safe and trustworthy deployment. Research focused on enhancing the transparency and robustness of LLMs will play a key role in addressing these concerns.
- **Simulation fidelity and generalization**. Despite the success of generative traffic models such as TrafficLLM and ChatSim in controlled environments, their performance in complex, real-world FAS networks with bursty interference or rare user scenarios remains limited. These models often struggle to generalize beyond the specific datasets they were trained on, particularly when exposed to unpredicted network conditions. Expanding and diversifying datasets, along with creating practical test beds that closely align with real-world FAS scenarios, is essential for improving the robustness and generalization of these models.
- **Standardization and evaluation of LLM-based agents**. Current LLM agent designs for FAS are still fragmented, with significant variation in architectural choices, interaction protocols, and tool integrations across different use cases. There is a pressing need for a unified framework that allows for the benchmarking, evaluation, and development of reusable, FAS-specific intelligent agents [62]. Establishing standardized evaluation criteria and fostering collaboration within the research community will help streamline progress in this area and enable fair comparisons across different approaches.

Looking forward, several promising research opportunities can be pursued to overcome the aforementioned challenges:

- **Hybrid optimization framework**. Integrating LLMs with black-box optimization search model, reinforcement learning, evolutionary algorithms, and AlphaEvolve-like approach can lead to the development of hybrid intelligence frameworks that improve both the adaptability and efficiency of FASs in dynamic environments;
- **Self-optimization, continuously evolving, autonomous agents**. Developing self-optimizing, continuously evolving autonomous agents, such as Auto-Agent for FAS, that can adapt in real-time based on feedback from the network environment will be crucial for building intelligent, autonomous systems capable of optimizing FAS operations without human intervention.
- **Generalizable simulation libraries and benchmark environments**. Constructing generalizable simulation libraries and benchmark environments specifically designed for FAS, 6G, and ISAC scenarios will provide a strong foundation for evaluating and comparing various traffic simulation models. These platforms will be critical for validating new techniques and ensuring their effectiveness in real-world deployments.
- **LLM-empowered FAS-ISAC**. Leveraging LLMs to enhance joint sensing and communication systems within FAS-ISAC networks offers significant potential for intelligent environmental perception, adaptive waveform optimization, and real-time data fusion. Such integrations will enable next-generation integrated networks that are more efficient, intelligent, and adaptable to the ever-changing network environment.

In addition to the technical challenges mentioned, the practical deployment of LLM-enhanced FAS faces critical issues related to model compression, fine-tuning, and domain adaptation, which require in-depth exploration for successful real-world applications. LLMs, while powerful, are often too large to be deployed effectively in resource-constrained environments such as edge devices. Model compression techniques [63], such as pruning, quantization, and knowledge distillation, offer potential solutions for reducing the size of these models without sacrificing performance. However, these approaches introduce challenges in maintaining accuracy, especially in dynamic FAS environments where real-time adaptation to user behavior is essential. Fine-tuning LLMs to specific network conditions and traffic scenarios is another avenue for improvement, enabling LLMs to perform optimally in specialized FAS contexts. This fine-tuning process requires continuous learning from real-world data, which adds complexity but is necessary for real-world deployment.

Another significant challenge lies in domain adaptation [64]. Most LLMs are pre-trained on generic datasets, which may not fully capture the diverse and dynamic nature of FAS network traffic. The ability of LLMs to adapt to specific domains, such as rural vs. urban environments or varying user behaviors in different regions, is crucial. Incorporating domain-specific data for training and fine-tuning LLMs ensures that models can generalize well across different FAS environments. This capability is particularly important for low-latency inference, where real-time decisions based on local network conditions are necessary. As FASs are often deployed in edge environments with stringent latency and computational resource limitations, optimizing computational efficiency is paramount. Research on lightweight models and edge-aware architectures is critical for ensuring that LLMs can provide real-time responses without excessive computational overhead. Techniques such as model distillation or parameter sharing across models could help mitigate the computational load while maintaining performance. Moreover, integrating LLMs with low-latency communication protocols will ensure that the system can adapt to rapid network changes, thereby improving the overall responsiveness of FAS in dynamic environments.

## 8. Conclusions

This survey examined the emerging role of LLMs in advancing FAS, demonstrating their effectiveness in overcoming key technical challenges. While these LLM-driven approaches show significant potential for optimizing channel estimation, antenna positioning, and network simulation, substantial work remains to address computational demands and ensure reliable deployment. The continued development of efficient algorithms and rigorous validation methods will be crucial for realizing practical implementations. As research progresses, the integration of artificial intelligence with reconfigurable antenna technology promises to transform future wireless communication systems.

## Figures and Tables

**Table 1:** Comparative evaluation of four LLM-based optimization methods. LLM-BB refers to LLM as the black-box optimization search model, LLM-DRL refers to LLM-guided deep reinforcement learning, LLM-EA refers to LLM-guided evolutionary algorithms, and LLM-Alpha refers to LLM as a solution program generator and critic; the maximum number of stars is 4.

	LLM-BB	LLM-DRL	LLM-EA	LLM-Alpha
Design difficulty	✩	✩✩	✩✩	✩✩✩
Solution quality	✩	✩✩✩✩	✩✩✩	✩✩✩✩
FLOP requirement	✩	✩✩✩	✩✩	✩✩✩✩
Memory requirement	✩	✩✩	✩✩	✩✩✩
Real time	✩✩	✩✩✩✩	✩✩	✩

**Table 2:** Three-path parameters for performance evaluation.

Parameter	Value	Parameter	Value
Path 1 Rx pitch angle, $\theta_{1}^{r}$	π/4	Path 1 Rx azimuth angle, $\theta_{1}^{t}$	π/3
Path 1 Tx pitch angle, $\phi_{1}^{r}$	π/6	Path 1 Tx azimuth angle, $\phi_{1}^{t}$	2π/5
Path 1 Rx pitch angle, $\theta_{2}^{r}$	π/5	Path 2 Rx azimuth angle, $\theta_{2}^{t}$	π/4
Path 1 Tx pitch angle, $\phi_{2}^{r}$	π/7	Path 2 Tx azimuth angle, $\phi_{2}^{t}$	π/7
Path 1 Rx pitch angle, $\theta_{3}^{r}$	π/3	Path 3 Rx azimuth angle, $\theta_{3}^{t}$	π/6
Path 1 Tx pitch angle, $\phi_{3}^{r}$	π/5	Path 3 Tx azimuth angle, $\phi_{3}^{t}$	π/4
Movable range	[−1,1] × [−1,1]	Path loss coefficient, α	2.5
Path 1 Gain, $\sigma_{1}$	0.634	Path 2 gain, $\sigma_{2}$	0.1768
Path 3 Gain, $\sigma_{3}$	0.1768	Frequency, *f*	60 GHz

## Data Availability

The original contributions presented in the study are included in the article. Further inquiries can be directed to the corresponding author.
